# Hyperforin, the major metabolite of St. John’s wort, exhibits pan-coronavirus antiviral activity

**DOI:** 10.3389/fmicb.2024.1443183

**Published:** 2024-08-08

**Authors:** Imelda Raczkiewicz, Céline Rivière, Peggy Bouquet, Lowiese Desmarets, Audrey Tarricone, Charline Camuzet, Nathan François, Gabriel Lefèvre, Fabiola Silva Angulo, Cyril Robil, François Trottein, Sevser Sahpaz, Jean Dubuisson, Sandrine Belouzard, Anne Goffard, Karin Séron

**Affiliations:** ^1^Univ. Lille, CNRS, Inserm, CHU Lille, Institut Pasteur de Lille, U1019 – UMR9017 – Center for Infection and Immunity of Lille (CIIL), Lille, France; ^2^BioEcoAgro, Joint Research Unit 1158, Univ. Lille, INRAE, Univ. Liège, UPJV, YNCREA, Univ. Artois, Univ. Littoral Côte d’Opale, ICV – Institut Charles Viollette, Lille, France

**Keywords:** coronaviruses, antiviral, natural compound, replication, broad-spectrum

## Abstract

**Introduction:**

The COVID-19 pandemic caused by the SARS-CoV-2 virus has underscored the urgent necessity for the development of antiviral compounds that can effectively target coronaviruses. In this study, we present the first evidence of the antiviral efficacy of hyperforin, a major metabolite of St. John’s wort, for which safety and bioavailability in humans have already been established.

**Methods:**

Antiviral assays were conducted in cell culture with four human coronaviruses: three of high virulence, SARS-CoV-2, SARS-CoV, and MERS-CoV, and one causing mild symptoms, HCoV-229E. The antiviral activity was also evaluated in human primary airway epithelial cells. To ascertain the viral step inhibited by hyperforin, time-of-addition assays were conducted. Subsequently, a combination assay of hyperforin with remdesivir was performed.

**Results:**

The results demonstrated that hyperforin exhibited notable antiviral activity against the four tested human coronaviruses, with IC_50_ values spanning from 0.24 to 2.55 µM. Kinetic studies indicated that the observed activity occur at a post-entry step, potentially during replication. The antiviral efficacy of hyperforin was additionally corroborated in human primary airway epithelial cells. The results demonstrated a reduction in both intracellular and extracellular SARS-CoV-2 viral RNA, confirming that hyperforin targeted the replication step. Finally, an additive antiviral effect on SARS-CoV-2 was observed when hyperforin was combined with remdesivir.

**Discussion:**

In conclusion, hyperforin has been identified as a novel pan-coronavirus inhibitor with activity in human primary airway epithelial cells, a preclinical model for coronaviruses. These findings collectively suggest that hyperforin has potential as a candidate antiviral agent against current and future human coronaviruses.

## 1 Introduction

The COVID-19 pandemic, caused by the severe acute respiratory syndrome coronavirus 2 (SARS-CoV-2), has emphasized the urgent need for broad-spectrum antiviral drugs. Before the emergence of SARS-CoV-2, there were no specific antiviral drugs available for human coronaviruses (HCoVs). Even 4 years after the onset of the outbreak, therapeutic options remain limited. To date, only three direct-acting antivirals have been approved by the Food and Drug Administration (FDA) for clinical use. Ritonavir-boosted nirmatrelvir (Paxlovid®) is the primary therapy for patients at high risk of developing severe COVID-19. Nirmatrelvir (PF-07321332) is an oral protease inhibitor that targets the main protease (M^pro^) of SARS-CoV-2 (Owen et al., 2021). Remdesivir, a viral RNA-dependent RNA polymerase (RdRp) inhibitor, is administered as a second-line therapy (Warren et al., 2016; Agostini et al., 2018; Beigel et al., 2020). Molnupiravir (MK-4482 or EIDD-2801), also an RdRp inhibitor, is an orally available prodrug of the ribonucleoside analog EIDD-1931 with broad-spectrum antiviral activity against RNA viruses (Stuyver et al., 2003; Sheahan et al., 2020).

Coronaviruses are positive single stranded-RNA viruses belonging to the *Coronaviridae* family, the *Orthocoronavirinae* subfamily and the Nidovirales order (Coronaviridae Study Group of the International Committee on Taxonomy of Viruses, 2020). A total of seven HCoV strains have been identified to date. These are distinguished into two groups based on their clinical presentation, from mild, HCoV-229E, -OC43, -HKU1, and-NL63, to severe symptoms, SARS-CoV, Middle-East respiratory syndrome coronavirus (MERS-CoV) and SARS-CoV-2 (Tang et al., 2022). Coronaviruses are enveloped RNA viruses. Structural proteins spike (S), envelope (E), and membrane (M) proteins are embedded into the envelope lipid bilayer and protect the viral genome which is associated with nucleoprotein (N). The S protein mediates the host-cell attachment and the viral entry by recognizing host-specific receptors (Belouzard et al., 2012). The virus enters into the cell via two different pathways, depending on the expression of cellular proteases on the cell surface, such as TMPRSS2 (Jackson et al., 2022). If the latter is expressed, the S protein is cleaved at the cell surface and the viral particle fuses with host plasma membrane. The endosomal pathway is the second entry pathway and the fusion occurs with the endosomal membrane (Hoffmann and Pöhlmann, 2021). The genome is then released into the cytosol, where it is translated into two polyproteins, pp1a and pp1ab. These two are cleaved by the papain-like protease nsp3 (PL^pro^) and the main protease nsp5 (M^PRO^), into several nonstructural proteins (nsp) such as nsp12, the viral RdRp, engaging the replication step (Weiss and Leibowitz, 2011). Then the virus is assembled and secreted.

Many SARS-Like CoVs have been described in bats, with some of them being capable of infecting human cells. Therefore, coronaviruses are still a threat for human health (Li et al., 2005; Temmam et al., 2022). Moreover, despite the widespread use of the current SARS-CoV-2 antiviral therapy, some concerns persist, such as the poor oral bioavailability of remdesivir (Rasmussen et al., 2022) and the mutagenic potency of molnupiravir (Zhou et al., 2021). The identification of new antiviral agents that could be utilized in combination therapies is essential to mitigate the emergence of resistant mutants and face future HCoV emergence.

Plants are a rich source of active molecules with a vast structural diversity which play a pivotal role in the field of drug discovery and serve as inspiration for medicinal chemistry (Newman and Cragg, 2016). Numerous plant metabolites are being identified as antimicrobials agents (Gibbons, 2004; Abreu et al., 2012). Among them, some products exhibit antiviral activity against viruses of different families *in vitro* (Denaro et al., 2020). Recently, pheophorbide a (Pba), a chlorophyll degradation product, and honokiol, isolated from magnolia bark, have demonstrated activity against several HCoVs (Meunier et al., 2022; Salgado-Benvindo et al., 2023). Here, we describe, for the first time, that hyperforin, the major metabolite of St. John’s wort (SJW), is an antiviral agent with pan-coronavirus activity.

## 2 Materials and methods

### 2.1 Chemicals and antibodies

Dulbecco’s modified Eagle’s medium (DMEM), phosphate-buffered saline (PBS), GlutaMAX™ and 4′, 6-diamidino-2-phenylindole (DAPI) were purchased from Life Technologies (Carlsbad, California, United States). Fetal bovine sera (FBS) were obtained from Eurobio (Evry, France). Remdesivir (GS-5734) and tariquidar were from BioTechne (Minneapolis, United States). GC376 was obtained from AmBeed (Arlington Heights, United States). Chloroquine and camostat mesylate were from Sigma-Aldrich (Saint Louis, United States). Hyperforin was purchased from Phytolab (Vestenbergsgreuth, Germany) (total of hyperforin >98.0%). Pba was from Cayman Chemicals (Merck Chemicals, Darmstadt, Germany). Stocks of compounds were resuspended in dimethyl sulfoxide (DMSO) at 100 mM.

Polyclonal rabbit anti-SARS-CoV-2 nucleocapsid antibody was purchased from Novus Biological (Cambridge, United Kingdom). Monoclonal mouse anti-ß-tubulin IgG1 antibody (T5201) was from Sigma-Aldrich (Saint Louis, United States). Horseradish peroxidase-conjugated goat anti-rabbit IgG antibody was purchased from Jackson ImmunoResearch (Ely, United Kingdom).

### 2.2 Cells

Human kidney cells (HEK293T/17, ATCC, CRL-11268; HEK293TT/ACE2) (Lavie et al., 2022), African green monkey kidney cells (Vero-81, ATCC, CCL-81; Vero-E6 cells), and Human hepatoma cells (Huh-7) were grown in DMEM supplemented with 10% FBS. Human lung cell line Calu-3 (ATCC, HTB-55) was cultivated in MEM supplemented with 10% FBS and glutaMAX-1. Lentiviral vectors expressing TMPRSS2 were used to transduce Vero-81 cells and to produce Huh-7/TMPRSS2 stable cell line. This latter was selected with 2 μg/mL of puromycin. A reporter cell line, F1G-Red, generated in the laboratory, and derivates from Vero-81 cells was also used for combination assay (Desmarets et al., 2022). Primary human nasal epithelia MucilAir™ (Epithelix, Geneva, Switzerland) were maintained in MucilAir™ culture medium (Epithelix) as recommended by the manufacturer.

### 2.3 Virus

HCoV-229E-luc was kindly gifted by Volker Thiel (van den Worm et al., 2012). SARS-CoV-2 variants the original strain containing the D614G mutation (EPI_ISL_940555) (Belouzard et al., 2022), the alpha (B1.1.7; EPI_ISL_1653931) and omicron variants (B1.1.529; EPI_ISL_7696645) provided by Dr. Kazali Alidjinou (University of Lille). SARS-CoV strain (Frankfurt isolate) (Drosten et al., 2003) was provided by Dr. Michelle Vialette (Unité de Sécurité Microbiologique, Pasteur Institute of Lille). MERS-CoV was recovered by transfecting the infectious clone of MERS-CoV-EMC12 (kindly provided by Luis Enjuanes) in Huh-7 cells (Almazán et al., 2013).

### 2.4 Cell viability

Huh-7, Vero-81 and Calu-3 cells were seeded in 96-well plate at a density of 1×10^4^, 1.5×10^4^, 5×10^4^ cells per well respectively, and incubated for 24 h at 37°C and 5% CO_2_ before treatment with the different compounds. Two-fold serial dilution were performed with final concentration ranging from 2.5 to 160 μM in DMEM. Cells were incubated with the compounds for 24 h. A 3-(4,5-dimethylthiazol-2-yl)-5-(3-carboxymethoxyphenyl)-2-(4-sulfophenyl)-2H-tetrazolium (MTS)-based viability assay (CellTiter 96® AQueous One Solution Cell Proliferation Assay, Promega) was performed as previously described (Meunier et al., 2022).

### 2.5 HCoVs infection assays

#### 2.5.1 HCoV-229E-Luc

2×10^4^ Huh-7/TMPRSS2 cells per well were seeded into a 96-well plate 24 h before infection. Cells were inoculated with HCoV-229E-Luc at a multiplicity of infection (MOI) of 0.1 and, simultaneously, treated with increasing concentrations of hyperforin (2-fold dilutions, from 0.625 to 20 μM). The inoculum was removed after 1 h and replaced with culture medium containing the compound at same concentrations. The cells were then lysed 7 h later in 20 μL of Renilla luciferase assay lysis buffer (Promega), and luciferase activity was quantified using a Tristar LB 941 luminometer (Berthold Technologies, Bad Bildbad, Germany) as recommended by the manufacturer.

#### 2.5.2 MERS-CoV and SARS-CoV

2×10^5^ Calu-3 or Vero-81/TMPRSS2 cells were seeded in a 24-well plate on coverslips, 48 h or 24 h prior infection with MERS-CoV or SARS-CoV, respectively. Cells were inoculated with the virus at a MOI of 0.1, in the presence of increased concentrations hyperforin (2-fold dilutions from 1.25 to 10 μM and 1.25 to 20 μM for SARS-CoV and MERS-CoV, respectively), for 1 h at 37°C and 5% of CO_2_. The inoculum was replaced by culture medium containing hyperforin at same concentrations and the cells were incubated for 16 h. Supernatants were collected for viral titration and cells were fixed twice with 4% of paraformaldehyde (PFA) before exiting the BSL-3 facility and processed for immunostaining.

#### 2.5.3 SARS-CoV-2

1×10^5^ Vero-81/TMPRSS2 cells per well were seeded in a 48-well plate 24 h before infection. Cells were inoculated with the virus at a MOI of 0.3, in the presence of increased concentration of the compound (2-fold dilutions from 0.625 to 10 μM), for 1 h at 37°C and 5% of CO_2_. 50 nM tariquidar, a P-glycoprotein inhibitor, was added in the media to inhibit pump efflux, and 10 μM chloroquine was used as a control of the expression of TMPRSS2. Inoculum was replaced with media containing the different compounds and cells were incubated for 16 h at 37°C and 5% of CO_2_. The supernatants were collected for viral titration.

### 2.6 Infectivity titration

Huh-7 (MERS-CoV) or Vero-E6 (SARS-CoV and SARS-CoV-2) were seeded in a 96-well plate and were inoculated with 1/10 serially diluted supernatants. After 5 days (SARS-CoV and SARS-CoV-2) or 7 days (MERS-CoV) of incubation at 37°C and 5% of CO_2_, the 50% tissue culture infectious dose (TCID50/mL) was determined by assessing the virus-induced cytopathic effect and using the Spearman-Kärber formula (Wulff et al., 2012).

### 2.7 Western blot detection

Proteins were separated onto a 12% SDS-polyacrylamide gel electrophoresis and transferred on nitrocellulose membrane (Hybond-ECL, Amersham). The membrane was blocked and incubated overnight at 4°C with a polyclonal rabbit anti-SARS-CoV-2 nucleocapsid antibody (1/4000), or a monoclonal mouse anti-ß-tubulin antibody (1/4000) and then with HRP-conjugated secondary antibodies. They were visualized by enhanced chemoluminescence (Pierce™ ECL, ThermoFisher Scientific) on LAS3000 (Fujifilm) or Amersham ImageQuant 800 (Cytiva).

### 2.8 MucilAir™ primary human airway epithelial cells (HAE) infection assay

The apical surface of the cells was rinsed 3 times for 10 min using MucilAir™ HAE culture medium to remove the mucosal secretion. The cells were inoculated at the apical side with HCoV-229E-Luc (MOI = 0.01) or SARS-CoV-2 (MOI = 0.3) and treated with 4 μM or 12 μM of hyperforin or 0.025% DMSO for 1 h. On the apical pole, the inoculum was removed and replaced by 10 μL of medium containing the compounds. Simultaneously, hyperforin or DMSO were added in the basolateral medium. For HCoV-229E-Luc infection, after 24 h of incubation, 140 μL of culture medium was added on the apical surface of MucilAir™ HAE and collected for RNA extraction. The cells were then lysed with 40 μL of Renilla luciferase assay lysis buffer (Promega). Luciferase activity was quantified as previously described. For SARS-CoV-2 infection, after 48 h of incubation, 140 μL of culture medium was added on the apical surface of MucilAir™ HAE and was collected for RNA quantification by RT-qPCR and viral titration. The cells were lysed and RNA was extracted for RT-qPCR assay.

### 2.9 RT-qPCR assay

RNA was extracted from MucilAir™-HAE supernatants or cells using QIAamp Viral RNA Mini kit (Qiagen) and NucleoSpin RNA plus (Macherey Nagel) respectively. One-step qPCR assay was performed using 5 μL of RNA and Takyon Low rox one-step RT probe master mix (UFD-LPRT-C0101, Eurogentec) with specific primers and probes (Supplementary Table S1) and using a Quantstudio 3 (Applied Biosystems). The expressions of HCoV-229E M gene and SARS-CoV-2 E gene were quantified using a standard curve.

### 2.10 Time-of-addition assay

One day prior infection, 1×10^5^ Vero-81/TMPRSS2 cells per well and 2×10^4^ Huh-7/TMPRSS2 cells per well were seeded into a 48-well plate or 96-well plate for SARS-CoV-2 or HCoV-229E-Luc infection, respectively. To assess which viral step is inhibited, the different compounds were added at different time points, either 1 h before the inoculation (corresponding to the condition “pre-treatment”), during the inoculation or 1 h, 2 h, 3 h after the inoculation (1 h.p.i., 2 h.p.i. or 3 h.p.i.). The cells were then lysed at 16 h or 7 h post-infection for SARS-CoV-2 or HCoV-229E-Luc, respectively, and analyzed as described above.

### 2.11 Drug combination assay

24 h before infection, 4.5×10^3^ F1G-Red cells per well were seeded in 384-well plate in DMEM with 2% of FBS. 1 h before infection, seven-2-fold concentrations of hyperforin and remdesivir combined or alone were dispensed onto the cells using an Echo 550 acoustic dispenser (Labcyte) in three biological replicates. DMSO concentration was kept constant throughout the plate and used as control. The cells were then inoculated with the virus by adding 10 μL of inoculum (MOI = 0.3) and 50 nM tariquidar for 16 h. The infection was assessed by using an INCELL Analyzer 6,500 high-throughput automated confocal microscope (GE Healthcare) located in BSL-3. Nine images were taken (20X objective, NA 0.75) for each condition and the number of infected cells was quantified using Columbus image analysis software (Perkin Elmer) (Desmarets et al., 2022). Synergy scores were calculated by using Synergy Finder (http://www.synergyfinderplus.org/) (mean and *p*-value).

### 2.12 Statistical analysis and IC_50_ and CC_50_ calculation

Analysis of the data was performed by using GraphPad Prism (Boston, Ma, United States). IC_50_ and CC_50_ values were calculated by nonlinear regression curve fitting with variable slopes and by constraining the top to 100% and the bottom to 0%. Results are presented as mean and standard error of the mean (SEM). Statistical analysis was performed using one-way Anova non-parametric Kruskal Wallis test followed with Dunn’s post-hoc test, and by comparing each treated group with the untreated control (DMSO control). *p*-values <0.05 were considered significantly different from the control.

## 3 Results

### 3.1 Hyperforin has pan-coronavirus activity

**Figure 1:**
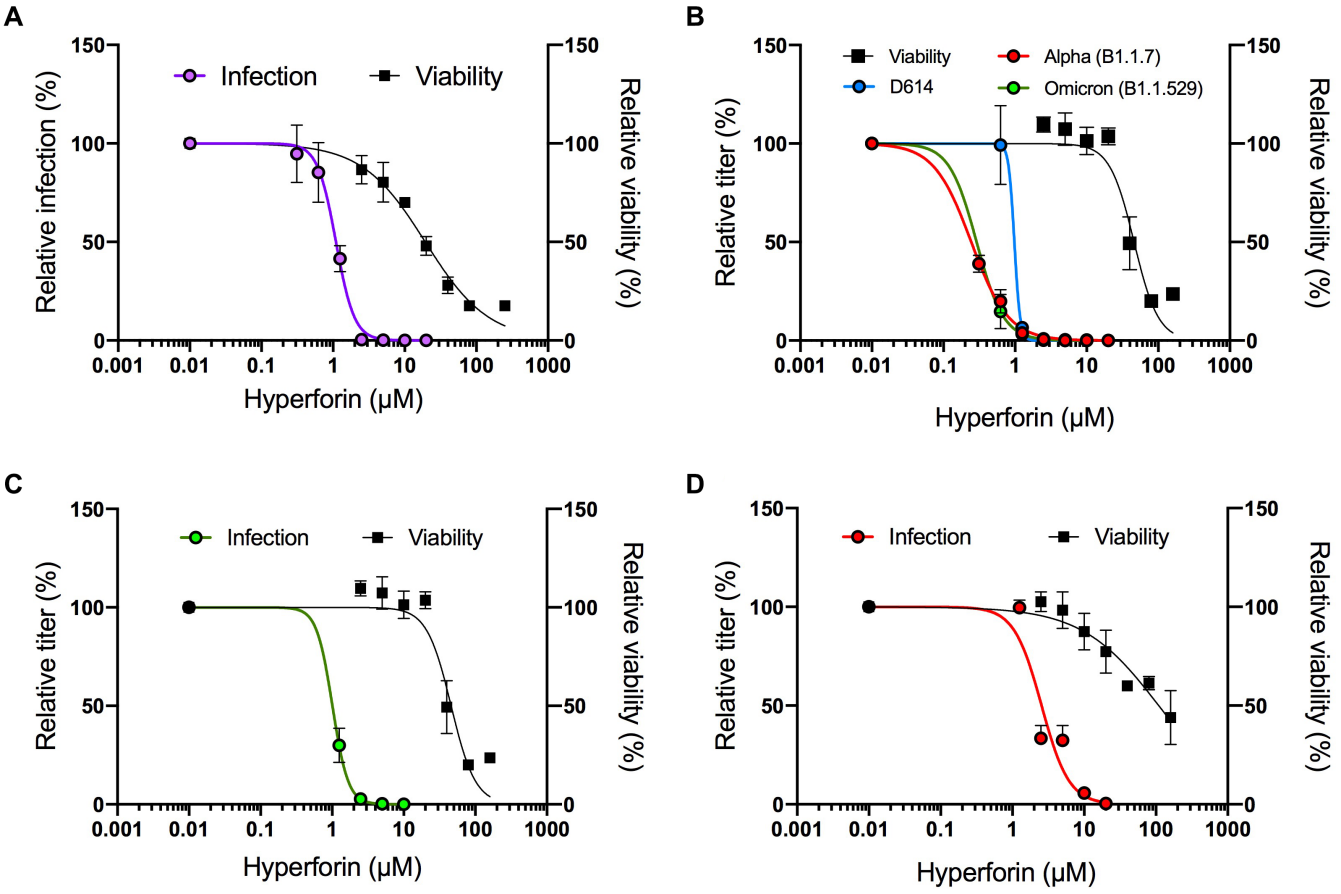
Hyperforin has a pan-coronavirus activity. Dose–response assays were performed against HCoV-229E-Luc in Huh-7/TMPRSS2 cells. Hyperforin concentrations are detailed in Materials and Methods section **(A)**, SARS-CoV-2 variants in Vero-81/TMPRSS2 **(B)**, SARS-CoV in Vero-81/TMPRSS2 **(C)** and MERS-CoV in Calu-3 cells **(D)**. Antiviral activity was studied either by measuring luciferase activity for HCoV-229E after 7 h of infection, or by TCID50/mL for SARS-CoV-2, SARS-CoV and MERS-CoV after 16 h of infection. Cytotoxicity was assessed by MTS assay at 24 h.

A preliminary screen of several natural compounds for their antiviral activity against HCoV-229E, a HCoV causing mild symptoms, identified hyperforin as a promising candidate. Indeed, dose–response assay showed an antiviral activity against HCoV-229E-Luc with a 50% inhibitory concentration (IC_50_) value of 1.10 μM ± 0.08 μM (Figure 1A). Cytotoxicity tests showed a 50% cytotoxic concentration (CC_50_) value of 19.35 ± 2.08 μM, resulting in a selective index (SI) of 17.59 (Table 1).

**Table 1:** Cytotoxicity and antiviral activity of hyperforin against HCoVs.

Virus	Cells	IC_50_ (μM)	CC_50_ (μM)	SI
HCoV-229E	Huh-7/TMPRSS2	1.10 ± 0.08	19.35 ± 2.08	17.59
SARS-CoV-2 (D614)	Vero-81/TMPRSS2	0.98 ± 0.28	45.91 ± 4.85	46.85
SARS-CoV-2 alpha (B1.1.7)	Vero-81/TMPRSS2	0.24 ± 0.02	45.91 ± 4.85	191.29
SARS-CoV-2 omicron (B1.1.529)	Vero-81/TMPRSS2	0.29 ± 0.13	45.91 ± 4.85	158.31
SARS-CoV	Vero-81/TMPRSS2	1.01 ± 0.12	45.91 ± 4.85	45.46
MERS-CoV	Calu-3	2.55 ± 0.28	≈ 100	≈ 39.21

We further investigated its antiviral activity against highly virulent HCoV. Dose–response assays were first conducted in cells challenged with SARS-CoV-2 variants D614G (B.1), alpha (B1.1.7) and omicron (B1.1.529). The results showed that hyperforin inhibited infection of the three variants with calculated IC_50_ values of 0.98 ± 0.28 μM, 0.24 ± 0.02 μM, and 0.29 ± 0.13 μM respectively, and with a CC_50_ value of 45.91 μM in Vero-81/TMPRSS2 cells (Figure 1B). The respective SI were all higher than 40 (Table 1). Importantly, antiviral activity of hyperforin was confirmed in human lung A549/ACE2 cells infected with SARS-CoV-2 (Supplementary Figures S1A,B). Next, dose–response assays were conducted to test hyperforin efficacy against SARS-CoV and MERS-CoV. The results highlighted that hyperforin is active against both of them with IC_50_ values of 1.01 ± 0.12 μM and 2.55 ± 0.28 μM, respectively (Figures 1C,D). These results were confirmed by immunostaining of double-stranded RNA for both virus and S protein of MERS-CoV (Supplementary Figures S1C–F). In Calu-3 cells, the CC_50_ value was estimated at approximately 100 μM (Figure 1D), which is consistent with the number of nuclei quantified by immunostaining (Supplementary Figure S1F). Taken together, these data demonstrated that hyperforin has a pan-coronavirus antiviral activity with IC_50_ values ranging from 0.24 to 2.55 μM.

### 3.2 Hyperforin inhibits a post-entry step

**Figure 2:**
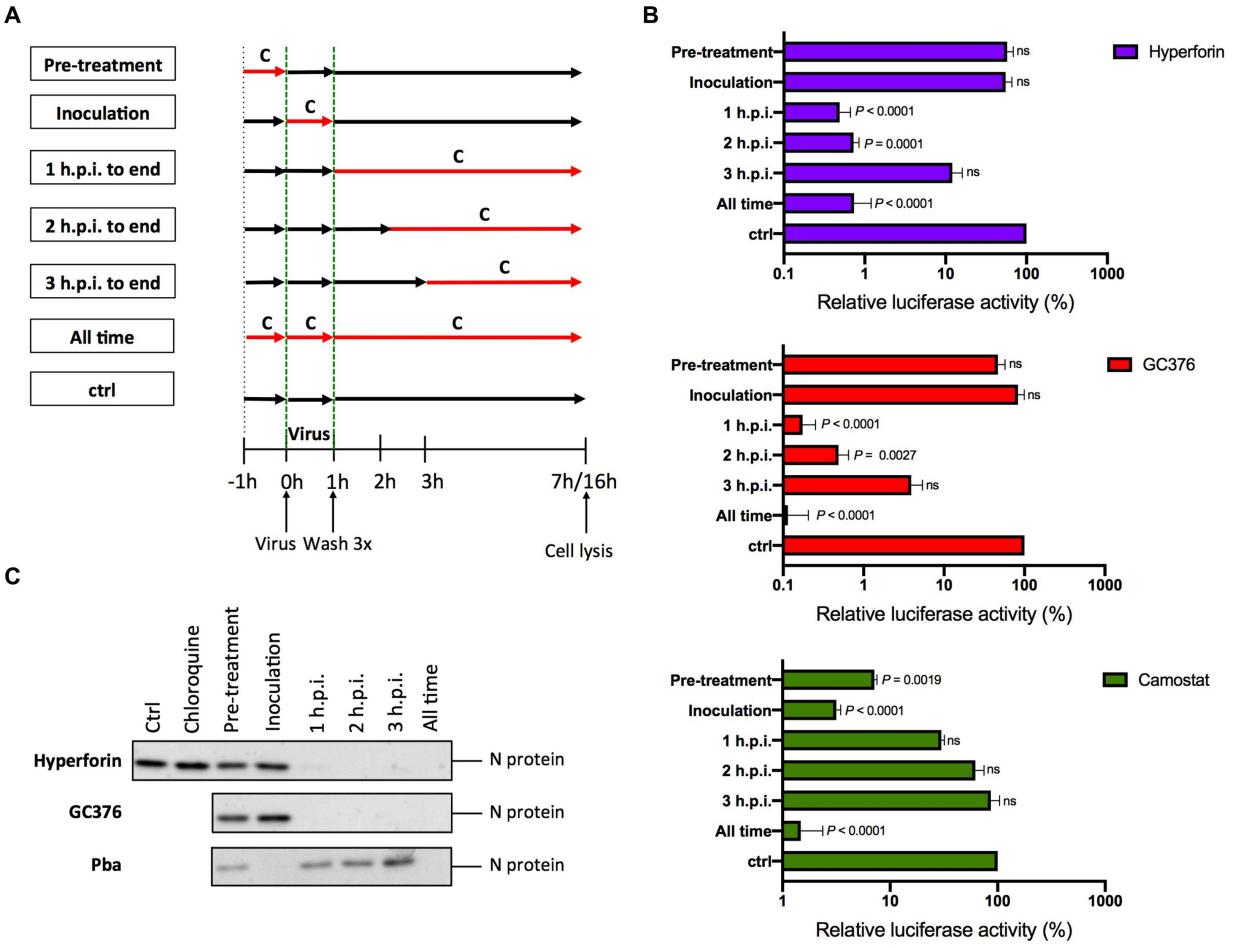
Hyperforin is active at a post entry step. **(A)** Graphical representation of the time-of-addition assay. Compounds **(C)** were added at different time points during infection. **(B)** 4 μM hyperforin, 5 μM GC376, 50 μM camostat mesylate (camostat) were added at different time points during infection of Huh-7/TMPRSS2 cells by HCoV-229E-Luc. **(C)** 20 μM hyperforin, 10 μM GC376 and 1 μM Pba, were added at different time points during infection of Vero-81/TMPRSS2 cells by SARS-CoV-2. 10 μM chloroquine was used as a control of TMPRSS2 expression. Cells were lysed 16 h after inoculation in Laëmmli loading buffer and the amount of N protein was detected in immunoblot.

To investigate the hyperforin mechanism of action, time-of-addition assays were performed (Figure 2A). The protease inhibitor GC376 and the TMPRSS2 inhibitor, camostat mesylate (entry inhibitor), were added as controls. Hyperforin significantly decreased the infection by more than 2Log_10_ from 1 h.p.i. to 2 h.p.i (Figure 2B). Similar kinetic profiles were observed for hyperforin and GC376 but not for camostat mesylate. These results were confirmed for SARS-CoV-2. N protein was not detected when hyperforin was added from 1 h.p.i. to 3 h.p.i (Figure 2C) and the N expression signal was quantified (Supplementary Figure S2). Similar inhibition profile to the SARS-CoV-2 Mpro inhibitor GC376 was observed. Conversely, hyperforin inhibition kinetic was different from the one of entry inhibitor Pba, a natural compound inhibiting SARS-CoV-2 entry (Meunier et al., 2022). To confirm that hyperforin is a post-entry inhibitor, assays were performed with particles pseudotyped with either HCoV-229E or SARS-CoV-2 spike protein (229 pp. or SARS2pp) mimicking virus entry. No significant decrease was observed for 229Epp or SARS2pp in the presence of hyperforin up to 5 μM (Supplementary Figure S3) suggesting that it is not an entry inhibitor. The data collectively indicate that hyperforin exerts its effects at a post-entry step of HCoV, most likely the replication step. Consequently, it is postulated that the compound may target a viral or cellular factor that is essential for the replication of the HCoV.

### 3.3 Hyperforin is active in primary human airway epithelial cells

**Figure 3:**
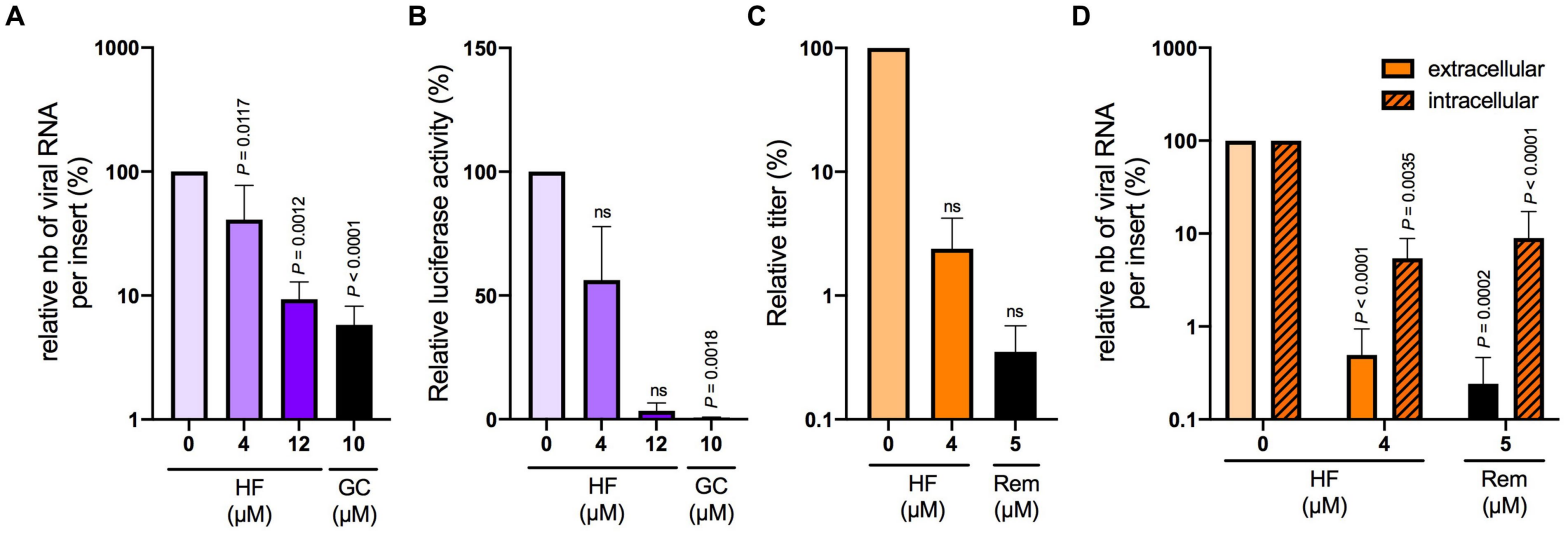
Hyperforin is active against HCoV-229E and SARS-CoV-2 in HAE.**(A)** Cells were inoculated with HCoV-229E-Luc at the apical surface of HAE in the presence of 4 or 12 μM hyperforin, and 10 μM GC376 (GC) for 24 h. Extracellular RNA was recovered from apical side and was quantified by RT-qPCR. **(B)** Cells were lysed and luciferase activity was measured. Cells were inoculated with SARS-CoV-2 at the apical surface in the presence of 4 μM hyperforin or 5 μM remdesivir (Rem) for 48 h. Infectious virus secreted at the apical surface was quantified by TCID50/mL **(C)** and intracellular and extracellular viral RNA by RT-qPCR analysis **(D)**.

To evaluate the potential use of hyperforin in HCoVs therapy, its antiviral activity was tested in HAE cultured at air-liquid interface, considered as a preclinical model for HCoVs. First, its cytotoxicity in HAE was determined by measuring LDH secretion and trans epithelial electrical resistance (TEER). LDH secretion higher than 5% and TEER lower than 100 Ω.cm^2^ reflect damaged cells. No cytotoxicity was observed with 4 μM hyperforin at 24 and 48 h for the two parameters (Supplementary Figure S4). However, cytotoxicity was observed with 12 μM hyperforin at 48 h, with LDH secretion higher than 5% compared to control and TEER lower than 100 Ω.cm^2^ (Supplementary Figure 4). Thus, for SARS-CoV-2 antiviral assays, only the concentration of 4 μM was evaluated due to an incubation time of 48 h. Antiviral assays with HCoV-229E-Luc in HAE showed a significant decrease in viral RNA copies with 12 μM hyperforin (Figures 3A,B), similar to the decrease observed with 10 μM GC376. No significant decrease of luciferase activity was observed at this concentration. For SARS-CoV-2, the data showed a decrease of infectious titers for both 4 μM hyperforin and 5 μM remdesivir used as control (Figure 3C). However, this decline was not statistically significant. Moreover, both intracellular and extracellular viral RNA are significantly decreased upon hyperforin treatment, similar to remdesivir control (Figure 3D), demonstrating that the reduction in virus release observed in Figure 3C is due to a reduction of viral RNA production. Taken together, these data underlined that hyperforin is active against HCoVs in HAE, a human preclinical model, and suggested that hyperforin is a replication inhibitor of SARS-CoV-2.

### 3.4 Hyperforin is active in combination with remdesivir

**Figure 4:**
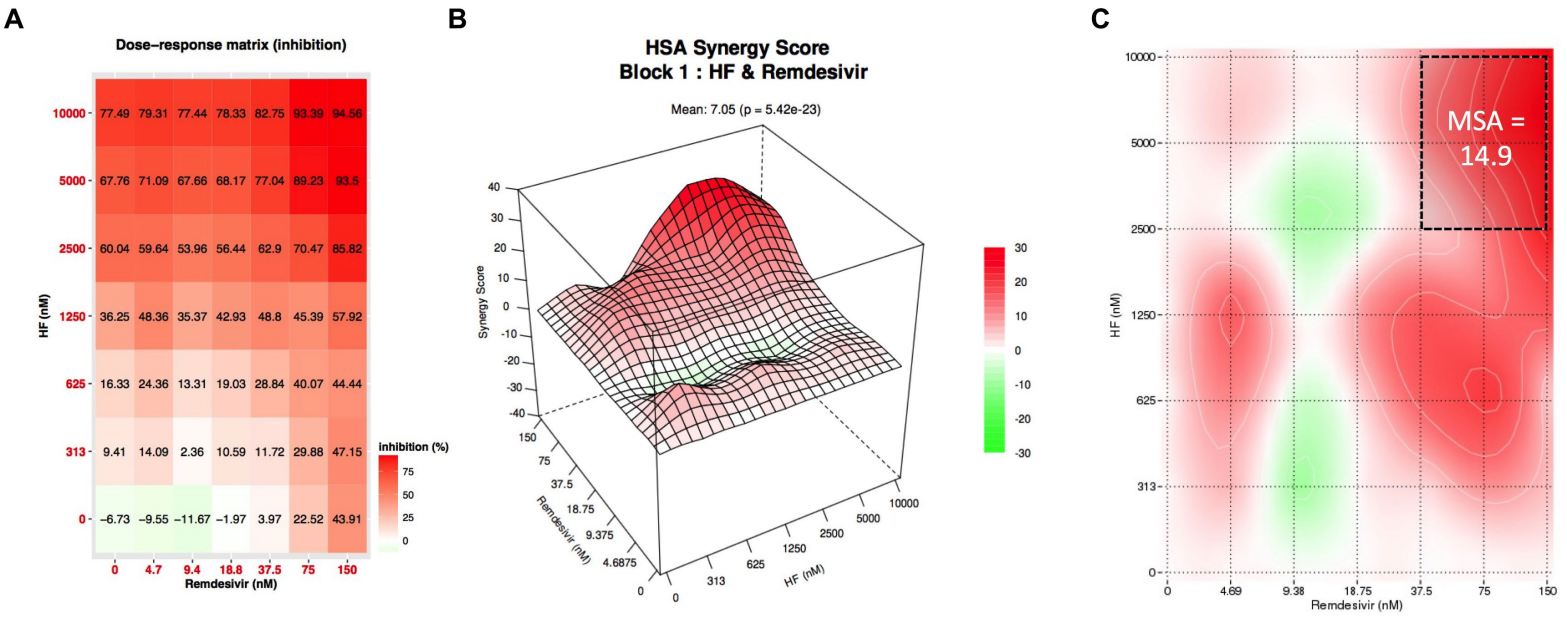
Combination of hyperforin and remdesivir. **(A)** Checkboard assay of hyperforin and remdesivir. **(B)** Inhibition of infection heatmap. **(C)** Most synergistic area (MSA) obtained with SynergyFinder 3.0 for HSA model.

The results presented above demonstrate that hyperforin encompasses many characteristics of an antiviral agent to be used in clinic. In order to reinforce the data, combination studies of hyperforin with remdesivir were performed using checkboard method with double serial dilutions of each compound (Figure 4A). Analysis by SynergyFinderPlus showed that the combination of hyperforin with remdesivir is additive, with synergy scores ranging from-10 to +10, with the four mathematical models (HSA, Loewe, Bliss and ZIP; Figure 4B and Supplementary Figure S5) and significant *p*-value (Table 2). Moreover, a most synergic area (MSA) analysis showed a synergistic combination with a MSA score of 14.9 (Figure 4C). Taken together, these results highlight the potential of hyperforin as an antiviral agent against HCoVs.

**Table 2:** Synergy scores obtained from synergy finder for hyperforin and remdesivir combination.

Model	Synergy score	*p*-value
ZIP	4.23	4.44e-04
Loewe	3.09	5.22e-03
HAS	7.05	5.42e-23
Bliss	5.13	9.11e-07

## 4 Discussion

This study presents, for the first time, evidence of the antiviral activity of hyperforin, a major metabolite of SJW (*Hypericum perforatum* L). Indeed, hyperforin exhibited antiviral activity against highly virulent human coronaviruses such as SARS-CoV, MERS-CoV, and SARS-CoV-2, as well as the low virulent HCoV-229E, with IC_50_ values spanning from nanomolar to micromolar ranges. Interestingly, pharmacokinetic and bioavailability data in mammals are already available for hyperforin due to the use of SJW extract for the treatment of mild to moderate depressive episodes (Barnes et al., 2001; Linde et al., 2008). Standardized extracts of SJW have been studied in many clinical trials demonstrating very good tolerability (Röder et al., 2004; Linde et al., 2008; EMA, 2023). Hyperforin, the major metabolite of SJW, represents up to 5% of the total extract (Barnes et al., 2001). Several studies have shown that circulating concentrations of hyperforin fall within the range of IC_50_ values, with maximal concentrations of 690 nM and 1.1 μM in rodents after administration of SJW extract or hyperforin (Biber et al., 1998; Hatanaka et al., 2011). In healthy human volunteers, ingestion of a single dose of 300 to 1,200 mg of SJW extract (containing 5% hyperforin) resulted in detectable concentrations of hyperforin in plasma ranging between 200 and 300 nM in two different studies (Biber et al., 1998; Agrosí et al., 2000; Hatanaka et al., 2011). Furthermore, various reports have demonstrated that SJW extract is not toxic in animal models and humans (Barnes et al., 2001; Negreş et al., 2016; EMA, 2023). Although these results are encouraging, the hyperforin distribution in lungs following an oral administration has not yet been reported. Donà et al. demonstrated that hyperforin could reduce lung metastases in mice after intraperitoneal injection, indicating that the compound can reach the lungs *in vivo* (Donà et al., 2004). Our results in HAE showed that hyperforin is active when administered at the air interface. It would be valuable to quantify hyperforin in mice lungs after intranasal administration. Pharmacokinetic studies are necessary to optimize the dose and route of administration.

Our findings indicate that hyperforin displays pan-coronavirus activity, suggesting that it may be efficacious against any known HCoV, as well as against any novel HCoVs that may emerge in the future. Few natural molecules have been reported to possess such activity, with the exceptions being Pba and honokiol (Meunier et al., 2022; Salgado-Benvindo et al., 2023). The identification of a pan-coronavirus antiviral drug represents a significant challenge in terms of pandemic preparedness (Jochmans et al., 2023). In this regard, hyperforin may potentially serve as a lead compound for the development of such an antiviral agent. It would be of interest to test the antiviral efficacy of hyperforin against the three other HCoVs, namely HCoV-OC43, -HKU1, and-NL63.

The results demonstrated that hyperforin is active at a post-entry step of HCoV, most likely during the replication step as it decreased both intracellular and extracellular RNA in HAE. Furthermore, the time-of-addition assay demonstrated a comparable inhibition profile for hyperforin and GC376, a protease inhibitor, which reinforces the hypothesis that hyperforin acts as a replication inhibitor. The replication step is a major target for antiviral agents employed in clinical settings, including nirmatrelvir and remdesivir. Consequently, it is of particular interest that hyperforin has also been demonstrated to target this step. To prevent viral resistance, it is widely accepted that antiviral therapy should combine two or three antiviral agents. Our results indicated that hyperforin could be combined with remdesivir, an RdRp inhibitor, showing additive antiviral activity and synergy at high concentrations *in vitro*. This combination is noteworthy as it was recently demonstrated that obeldesivir, an oral prodrug of the parent remdesivir was shown to be very effective in non-human primates (Mackman et al., 2023).

Further experiments are required in order to gain a more comprehensive understanding of the precise mechanism of action of hyperforin. For instance, transcriptomic and proteomic analyses may provide insight into the cellular pathways that are modulated by hyperforin. Hyperforin has recently been described as an inducer of the heme oxygenase 1 (HO-1) pathway, with the ability to upregulate the expression of HO-1 (Cardile et al., 2023). Moreover, it has been demonstrated that hemin, an inducer of HO-1 that upregulates HO-1 expression, exhibits antiviral activity against SARS-CoV-2 (Kim et al., 2021). In our study, it was not possible to demonstrate the upregulation of HO-1 expression in response to hyperforin treatment in either the Huh-7 or Vero-81 cell lines (Supplementary Figure S6A). Moreover, the down-regulation of HO-1 expression by specific siRNA did not show an impact on HCoV-229E infection (Supplementary Figures S6B,C), suggesting that HO-1 is not involved in antiviral activity of hyperforin.

As mentioned previously, hyperforin is the major metabolite of SJW extract. *Hypericum perforatum* L. (SJW), is the most common and well-known species in the genus Hypericaceae. Consequently, hyperforin is potentially available in large quantities. Further *in vivo* investigation, in mouse model, is required to substantiate the potential of hyperforin as a pan-coronavirus antiviral agent. It is noteworthy that it has already been demonstrated that this compound is orally available.

In conclusion, the imperative for broad-spectrum antiviral agents has become increasingly evident to bolster pandemic preparedness and address the emergence of future HCoVs. Given the existing clinical reports and the antiviral efficacy demonstrated in this study, hyperforin holds considerable promise for future therapies targeting both human and animal coronaviruses.

## Data availability statement

The original contributions presented in the study are included in the article/Supplementary material, further inquiries can be directed to the corresponding authors.

## Author contributions

IR: Writing – original draft, Formal analysis, Investigation, Methodology, Writing – review & editing. CRi: Conceptualization, Writing – review & editing. PB: Investigation, Writing – review & editing. LD: Formal Analysis, Investigation, Methodology, Writing – review & editing. AT: Investigation, Writing – review & editing. CC: Investigation, Writing – review & editing. NF: Investigation, Writing – review & editing. GL: Methodology, Writing – review & editing. FS: Investigation, Writing – review & editing. CRo: Investigation, Writing – review & editing. FT: Methodology, Writing – review & editing. SS: Conceptualization, Writing – review & editing. JD: Funding acquisition, Writing – review & editing. SB: Conceptualization, Funding acquisition, Writing – review & editing. AG: Methodology, Writing – review & editing. KS: Conceptualization, Formal analysis, Funding acquisition, Methodology, Writing – original draft, Writing – review & editing.

## References

[ref1] Abreu A. C. McBain A. J. Simões M. (2012). Plants as sources of new antimicrobials and resistance-modifying agents. Nat. Prod. Rep. 29, 1007–1021. doi: 10.1039/c2np20035j, PMID: 22786554

[ref2] Agostini M. L. Andres E. L. Sims A. C. Graham R. L. Sheahan T. P. Xiaotao L.. (2018). Coronavirus susceptibility to the antiviral Remdesivir (GS-5734) is mediated by the viral polymerase and the proofreading exoribonuclease. MBio 9:e00221-18. doi: 10.1128/mbio.00221-18 29511076 PMC5844999

[ref3] Agrosí M. Mischiatti S. Harrasser P. C. Savio D. (2000). Oral bioavailability of active principles from herbal products in humans. A study on *Hypericum Perforatum* extracts using the soft gelatin capsule technology. Phytomedicine 7, 455–462. doi: 10.1016/S0944-7113(00)80029-X, PMID: 11194173

[ref4] Almazán F. DeDiego M. L. Sola I. Zuñiga S. Nieto-Torres J. L. Marquez-Jurado S.. (2013). Engineering a replication-competent, propagation-defective Middle East respiratory syndrome coronavirus as a vaccine candidate. Am. Soc. Microbiol. 4, e00650–e00613. doi: 10.1128/mBio.00650-13, PMID: 24023385 PMC3774192

[ref5] Barnes J. Anderson L. A. Phillipson J. D. (2001). St John’s wort (*Hypericum Perforatum* L.): a review of its chemistry, pharmacology and clinical properties. J. Pharm. Pharmacol. 53, 583–600. doi: 10.1211/0022357011775910, PMID: 11370698

[ref6] Beigel J. H. Tomashek K. M. Dodd L. E. Mehta A. K. Zingman B. S. Kalil A. C.. (2020). Remdesivir for the treatment of Covid-19- final report. N. Engl. J. Med. 383, 1813–1826. doi: 10.1056/NEJMoa2007764, PMID: 32445440 PMC7262788

[ref7] Belouzard S. Machelart A. Sencio V. Vausselin T. Hoffmann E. Deboosere N.. (2022). Clofoctol inhibits SARS-CoV-2 replication and reduces lung pathology in mice. PLoS Pathog. 18:e1010498. doi: 10.1371/journal.ppat.1010498, PMID: 35587469 PMC9119441

[ref8] Belouzard S. Millet J. K. Licitra B. N. Whittaker G. R. (2012). Mechanisms of coronavirus cell entry mediated by the viral spike protein. Viruses 4, 1011–1033. doi: 10.3390/v4061011, PMID: 22816037 PMC3397359

[ref9] Biber A. Fischer H. Römer A. Chatterjee S. S. (1998). Oral bioavailability of Hyperforin from Hypericum extracts in rats and human volunteers. Pharmacopsychiatry 31, 36–43. doi: 10.1055/s-2007-979344, PMID: 9684946

[ref10] Cardile A. Passarini C. Zanrè V. Fiore A. Menegazzi M. (2023). Hyperforin enhances Heme Oxygenase-1 expression triggering lipid peroxidation in BRAF-mutated melanoma cells and hampers the expression of pro-metastatic markers. Antioxidants 12:1369. doi: 10.3390/antiox12071369 37507910 PMC10376533

[ref11] Coronaviridae Study Group of the International Committee on Taxonomy of Viruses (2020). The species severe acute respiratory syndrome-related coronavirus: classifying 2019-nCoV and naming it SARS-CoV-2. Nat. Microbiol. 5, 536–544. doi: 10.1038/s41564-020-0695-z, PMID: 32123347 PMC7095448

[ref12] Denaro M. Smeriglio A. Barreca D. De Francesco C. Occhiuto C. Milano G.. (2020). Antiviral activity of plants and their isolated bioactive compounds: an update. Phytother. Res. 34, 742–768. doi: 10.1002/ptr.6575 31858645

[ref13] Desmarets L. Callens N. Hoffmann E. Danneels A. Lavie M. Couturier C.. (2022). A reporter cell line for the automated quantification of SARS-CoV-2 infection in living cells. Front. Microbiol. 13:1031204. doi: 10.3389/fmicb.2022.1031204, PMID: 36246297 PMC9558224

[ref14] Donà M. Dell’Aica I. Pezzato E. Sartor L. Calabrese F. Barbera M. D.. (2004). Hyperforin inhibits Cancer invasion and metastasis. Cancer Res. 64, 6225–6232. doi: 10.1158/0008-5472.CAN-04-0280, PMID: 15342408

[ref15] Drosten C. Günther S. Preiser W. van der Werf S. Brodt H.-R. Becker S.. (2003). Identification of a novel coronavirus in patients with severe acute respiratory syndrome. N. Engl. J. Med. 348, 1967–1976. doi: 10.1056/NEJMoa030747, PMID: 12690091

[ref16] EMA. (2023). “Hyperici Herba.” Text. European Medicines Agency. Available at: https://www.ema.europa.eu/en/medicines/herbal/hyperici-herba-0 (Accessed February 22, 2023).

[ref17] Gibbons S. (2004). Anti-staphylococcal plant natural products. Nat. Prod. Rep. 21, 263–277. doi: 10.1039/b212695h 15042149

[ref18] Hatanaka J. Shinme Y. Kuriyama K. Uchida A. Kou K. Uchida S.. (2011). In vitro and in vivo characterization of new formulations of St. John’s wort extract with improved pharmacokinetics and anti-nociceptive effect. Drug Metab. Pharmacokinet. 26, 551–558. doi: 10.2133/dmpk.DMPK-11-RG-041, PMID: 21914965

[ref19] Hoffmann M. Pöhlmann S. (2021). How SARS-CoV-2 makes the cut. Nat. Microbiol. 6, 828–829. doi: 10.1038/s41564-021-00931-x 34194035

[ref20] Jackson C. B. Farzan M. Chen B. Choe H. (2022). Mechanisms of SARS-CoV-2 entry into cells. Nat. Rev. Mol. Cell Biol. 23, 3–20. doi: 10.1038/s41580-021-00418-x, PMID: 34611326 PMC8491763

[ref21] Jochmans D. Laporte M. Neyts J. (2023). Antiviral strategies for epidemic and pandemic preparedness. Cell Host Microbe 31, 856–860. doi: 10.1016/j.chom.2023.05.012, PMID: 37321170

[ref22] Kim D.-H. Ahn H.-S. Go H.-J. Kim D.-Y. Kim J.-H. Lee J.-B.. (2021). Hemin as a novel candidate for treating COVID-19 via Heme Oxygenase-1 induction. Sci. Rep. 11:21462. doi: 10.1038/s41598-021-01054-3, PMID: 34728736 PMC8563742

[ref23] Lavie M. Dubuisson J. Belouzard S. (2022). SARS-CoV-2 spike Furin cleavage site and S2’ basic residues modulate the entry process in a host cell-dependent manner. J. Virol. 96:e0047422. doi: 10.1128/jvi.00474-22, PMID: 35678602 PMC9278140

[ref24] Li W. Shi Z. Meng Y. Ren W. Smith C. Epstein J. H.. (2005). Bats are natural reservoirs of SARS-like coronaviruses. Science 310, 676–679. doi: 10.1126/science.1118391 16195424

[ref25] Linde K. Berner M. M. Kriston L. (2008). St John’s wort for major depression. Cochrane Database Syst. Rev. 2008:CD000448. doi: 10.1002/14651858.CD000448.pub3 18843608 PMC7032678

[ref26] Mackman R. L. Kalla R. V. Babusis D. Pitts J. Barrett K. T. Chun K.. (2023). Discovery of GS-5245 (Obeldesivir), an Oral prodrug of nucleoside GS-441524 that exhibits antiviral efficacy in SARS-CoV-2-infected African Green monkeys. J. Med. Chem. 66, 11701–11717. doi: 10.1021/acs.jmedchem.3c00750 37596939 PMC11556372

[ref27] Meunier T. Desmarets L. Bordage S. Bamba M. Hervouet K. Rouillé Y.. (2022). A Photoactivable natural product with broad antiviral activity against enveloped viruses, including highly pathogenic coronaviruses. Antimicrob. Agents Chemother. 66:e0158121. doi: 10.1128/AAC.01581-21, PMID: 34807755 PMC8846325

[ref28] Negreş S. Scutari C. Ionică F. E. Gonciar V. Velescu B. Ş. Şeremet O. C.. (2016). Influence of Hyperforin on the morphology of internal organs and biochemical parameters, in experimental model in mice. Rom. J. Morphol. Embryol. 57, 663–673, PMID: 27833957

[ref29] Newman D. J. Cragg G. M. (2016). Natural products as sources of new drugs from 1981 to 2014. J. Nat. Prod. 79, 629–661. doi: 10.1021/acs.jnatprod.5b01055 26852623

[ref30] Owen D. R. Allerton C. M. N. Anderson A. S. Aschenbrenner L. Avery M. Berritt S.. (2021). An Oral SARS-CoV-2 Mpro inhibitor clinical candidate for the treatment of COVID-19. Science 374, 1586–1593. doi: 10.1126/science.abl4784 34726479

[ref31] Rasmussen H. B. Thomsen R. Hansen P. R. (2022). Nucleoside analog GS-441524: pharmacokinetics in different species, safety, and potential effectiveness against Covid-19. Pharmacol. Res. Perspect. 10:e00945. doi: 10.1002/prp2.945, PMID: 35396928 PMC8994193

[ref32] Röder C. Schaefer M. Leucht S. (2004). Meta-analysis of effectiveness and tolerability of treatment of mild to moderate depression with St. John’s wort. Fortschr. Neurol. Psychiatr. 72, 330–343. doi: 10.1055/s-2003-812513, PMID: 15211398

[ref33] Salgado-Benvindo C. Leijs A. A. Thaler M. Tas A. Arbiser J. L. Snijder E. J.. (2023). Honokiol inhibits SARS-CoV-2 replication in cell culture at a post-entry step. Microbiol. Spectr. 11:e0327322. doi: 10.1128/spectrum.03273-22, PMID: 37212560 PMC10269499

[ref34] Sheahan T. P. Sims A. C. Zhou S. Graham R. L. Pruijssers A. J. Agostini M. L.. (2020). An orally bioavailable broad-Spectrum antiviral inhibits SARS-CoV-2 in human airway epithelial cell cultures and multiple coronaviruses in mice. Sci. Transl. Med. 12:eabb5883. doi: 10.1126/scitranslmed.abb5883 32253226 PMC7164393

[ref35] Stuyver L. J. Whitaker T. McBrayer T. R. Hernandez-Santiago B. I. Lostia S. Tharnish P. M.. (2003). Ribonucleoside analogue that blocks replication of bovine viral diarrhea and hepatitis C viruses in culture. Antimicrob. Agents Chemother. 47, 244–254. doi: 10.1128/aac.47.1.244-254.2003, PMID: 12499198 PMC149013

[ref36] Tang G. Liu Z. Chen D. (2022). Human coronaviruses: origin, host and receptor. J. Clin. Virol. 155:105246. doi: 10.1016/j.jcv.2022.105246, PMID: 35930858 PMC9301904

[ref37] Temmam S. Vongphayloth K. Baquero E. Munier S. Bonomi M. Regnault B.. (2022). Bat coronaviruses related to SARS-CoV-2 and infectious for human cells. Nature 604, 330–336. doi: 10.1038/s41586-022-04532-4, PMID: 35172323

[ref38] Warren T. K. Jordan R. Lo M. K. Ray A. S. Mackman R. L. Soloveva V.. (2016). Therapeutic efficacy of the small molecule GS-5734 against Ebola virus in Rhesus monkeys. Nature 531, 381–385. doi: 10.1038/nature17180, PMID: 26934220 PMC5551389

[ref39] Weiss S. R. Leibowitz J. L. (2011). Coronavirus pathogenesis. Adv. Virus Res. 81, 85–164. doi: 10.1016/B978-0-12-385885-6.00009-2, PMID: 22094080 PMC7149603

[ref40] van den Worm S. H. E. Eriksson K. K. Zevenhoven J. C. Weber F. Züst R. Kuri T.. (2012). Reverse genetics of SARS-related coronavirus using vaccinia virus-based recombination. PLoS One 7:e32857. doi: 10.1371/journal.pone.0032857, PMID: 22412934 PMC3296753

[ref41] Wulff N. H. Tzatzaris M. Young P. J. (2012). Monte Carlo simulation of the spearman-Kaerber TCID50. J. Clin. Bioinform. 2:5. doi: 10.1186/2043-9113-2-5, PMID: 22330733 PMC3331834

[ref42] Zhou S. Hill C. S. Sarkar S. Tse L. V. Woodburn B. M. D. Schinazi R. F.. (2021). β-D-N4-Hydroxycytidine inhibits SARS-CoV-2 through lethal mutagenesis but is also mutagenic to mammalian cells. J. Infect. Dis. 224, 415–419. doi: 10.1093/infdis/jiab247, PMID: 33961695 PMC8136050

