# Supplementary material for: Hyperforin, the major metabolite of St. John’s wort, exhibits pan-coronavirus antiviral activity
Source: Front Microbiol. 2024 Aug 8;15:1443183. doi: 10.3389/fmicb.2024.1443183 (PMC11339956; doi:10.3389/fmicb.2024.1443183)
Supplement: Supplementary file 1 [file Data_Sheet_1.pdf]

frontiers

Supplementary Material

1 Supplementary material

1.1 Chemicals and antibodies

Hemin was from Sigma-Aldrich (Saint Louis, USA). Mouse anti-dsRNA mAb (clone J2) was from Scicons (Nordic-MUbio, Susteren, the Netherlands). Rabbit polyclonal anti-MERS-CoV Spike protein antibody was from SinoBiological (Eschborn, Germany). Alexa 594-conjugated goat anti-rabbit antibody and Alexa 488-conjugated donkey anti-mouse antibody, were purchased from Jackson ImmunoResearch (Ely, United Kingdom).

1.2 SARS-CoV-2 infection in A549/ACE2 cells

Human lung cells (A549/ACE2, kindly provided by Delphine Muriaux) were grown in DMEM supplemented with 10% FBS. $1x10^5$ A549/ACE2 cells per well were seeded in a 48-well plate 24 h before infection. Cells were inoculated with the virus at a MOI of 0.3, in the presence of increased concentration of the compound, for 1 h at 37°C and 5% of CO_2. Inoculum was replaced with media containing the different compounds and cells were incubated for 16 h at 37°C and 5% of CO_2. Cells were lysed using reducing Laëmmli loading buffer for western blot analysis. The samples were inactivated 30 min at 95°C.

1.3 Immunostaining

Cells were permeabilized for 5 min with 0.4 % Triton X-100 and blocked with 5% of goat serum for 30 min and were incubated with anti-dsRNA monoclonal mouse antibody (clone J2) or anti-MERS-CoV Spike protein polyclonal rabbit antibody. Cells were rinsed three times with PBS, and immunostained with an Alexa 594-conjugated goat anti-rabbit secondary antibody or an alexa-488-conjugated donkey anti-mouse secondary antibody and DAPI. The coverslips were mounted on microscope slides in Mowiol. The images were acquired with an Evos M5000 microscope (Thermo Fisher Scientific). Ten images were randomly taken for each condition in duplicate. The number of cells were determined by the number of nuclei, and infected cells were detected by quantifying the number of dsRNA-positive or Spike-positive cells using ImageJ software.

1.4 Pseudotyped particle entry assay

Particles pseudotyped with either the spike protein of SARS-CoV-2 (SARS2pp), or HCoV-229E (229Epp), were produced as previously described (Belouzard, Chu, and Whittaker 2009). $4.5x10^3$ HEK293TT/ACE2 (SARS2pp) cells per well or $1x10^4$ Huh-7/TMPRSS2 (229Epp) cells per well were seeded into a 96-well plate 24 h before infection. Cells were inoculated with SARS2pp and 229Epp in the presence of hyperforin (2.5, 5 or 10 μM) for 2 h at 37°C and 5% of CO_2. The inoculum was removed and replaced with culture media without compound. The cells were lysed after 48 h of incubation. The

luciferase activity was then measured with Firefly luciferase assay kit (Promega) according to the manufacturer recommendations and using a luminometer (Berthold).

1.5 MucilAir™ cytoxicity assays

Cytotoxicity was studied according to the manufacturer's instruction either using cytotoxicity lactate deshydrogenase (LDH) assay kit-WST (Dojindo) or by measuring transepithelial electrical resistance (TEER) (Millicell® ERS-2, Millipore) as previously described (Meunier et al. 2022). Toxicity is considered when LDH secretion is above 5% and TEER below 100 $\Omega.cm^2$.

1.6 RNA interference

Cells were transfected with small interfering RNA (siRNA) targeting HO-1 (siHO-1, final concentration: 10 nM) (Gene HMOX1, AM16708, Assay ID 11056, ThermoFisher) or with a non-targeting siRNA control (siCTRL, Dharmacon) using Lipofectamine RNAi MAX (Invitrogen) according to manufacturer's recommendations. After 24 h of incubation, cells were treated with 50µM of hemin or increasing concentration of hyperforin (2-fold dilutions, from 1.25 to 10 µM), infected or not with HCoV-229E-Luc and incubated again for 24 h. Cells were then lysed using Laëmmli buffer in reducing conditions or with the Renilla luciferase assay lysis buffer. HO-1 expression was then studied by Western Blot.

2 Supplementary Figures and Tables

2.1 Supplementary Figures

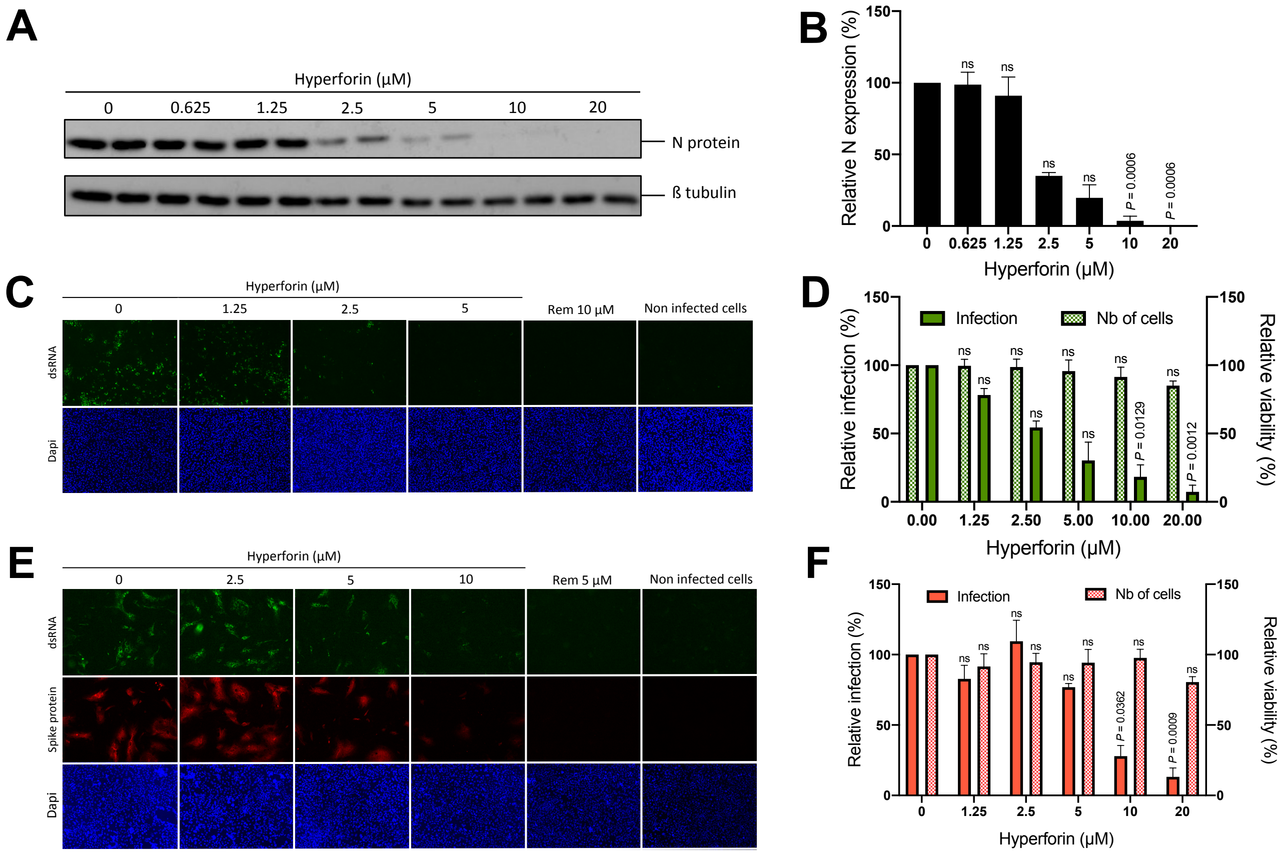

Supplementary Figure 1. Hyperforin is active on SARS-CoV-2, SARS-CoV and MERS-CoV
(A) Human lung A549/ACE2 cells were infected with SARS-CoV-2 alpha variant in the presence of increasing concentrations of hyperforin for 16 h. Cells were lysed and Western Blot analysis was performed using anti-N and anti-tubulin antibodies. Western blot is representative of 3 independent experiments. **(B)** Western blot quantification of N expression signal. **(C)** Vero-81/TMPRSS2 cells were inoculated with SARS-CoV for 16 h. Double stranded RNA was detected by immunofluorescence and nuclei were stained with DAPI. (**D**) Total cell number and SARS-CoV infected cells were quantified using ImageJ software. **(E)** Calu-3 cells were inoculated with MERS-CoV for 16 h. Double stranded RNA and MERS-CoV spike protein were detected by immunofluorescence and nuclei were stained with DAPI. (**F**) Total cell number and MERS-CoV infected cells were quantified by ImageJ software. Images are representative of 3 independent experiments.

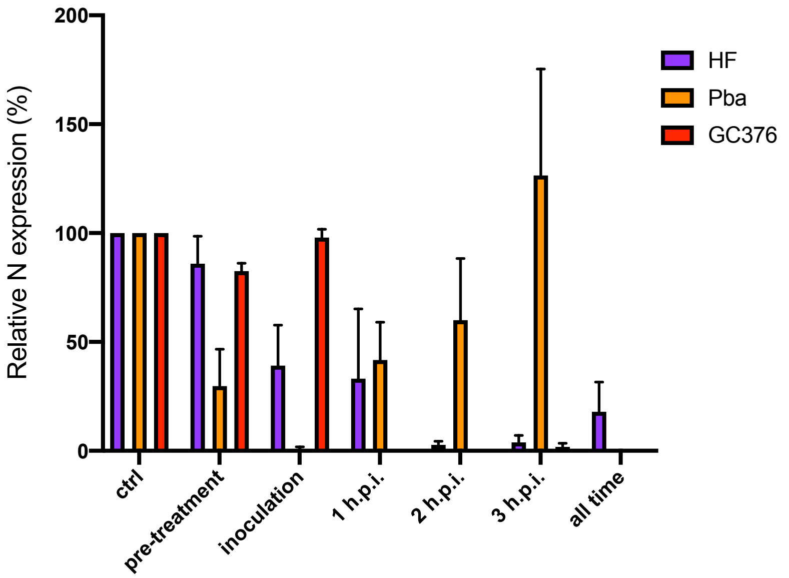

Supplementary Figure 2. Quantification of N expression in time-of-addition assay.

20 µM hyperforin, 10 µM GC376 and 1 µM Pba, were added at different time points (see **Figure 2A**) during infection of Vero-81/TMPRSS2 cells by SARS-CoV-2. Cells were lysed 16 h after inoculation in Laëmmli loading buffer and the amount of N protein was detected in immunoblot. The signal was quantified using ImageJ software and expressed relative to the control without compound.

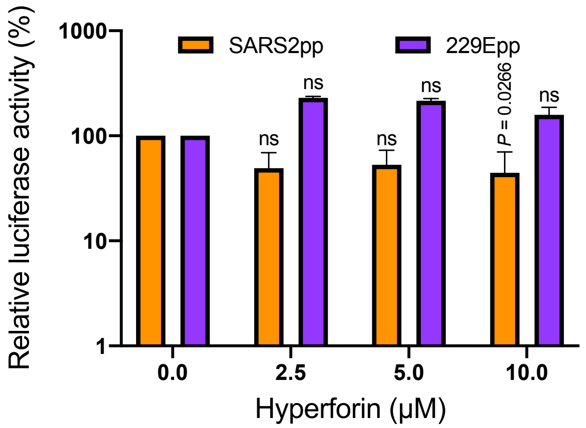

Supplementary Figure 3. Hyperforin is not an entry inhibitor.

HEK-293TT/ACE2 and Huh-7/TMPRSS2 cells were inoculated with SARSpp and 229Epp, respectively in the presence of increasing concentrations of hyperforin for 2 h. Inoculum was removed and replaced with medium without compound for 46 h. Cells were lysed and luciferase activity was quantified. Data were expressed as mean +/- SEM of 3 independent experiments performed in triplicate. Statistical analyses were performed using the Mann-Whitney nonparametric test. n.s. not significant.

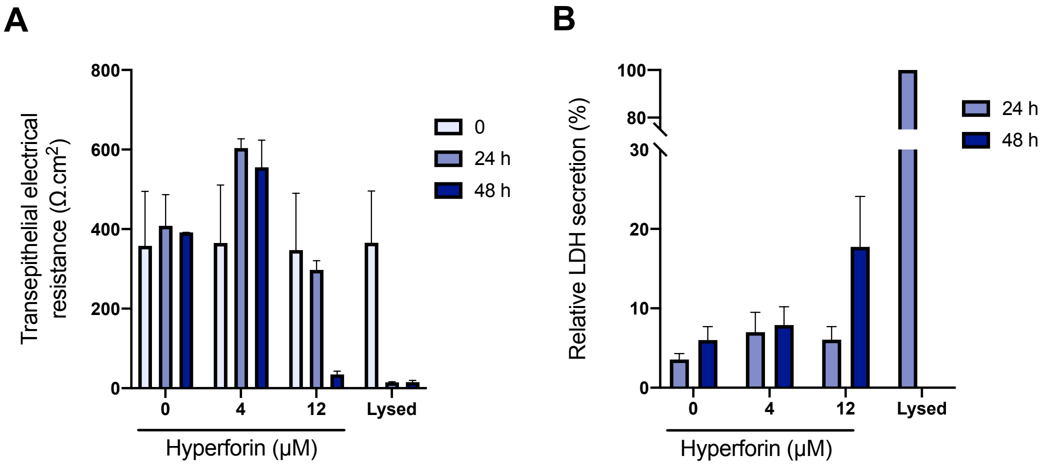

Supplementary Figure 4. Evaluation of hyperforin cytotoxicity in HAE cells.
Cytotoxicity of hyperforin was determined at 24 h and 48 h by measuring the TEER (**A**) and LDH secretion (**B**) according to the manufacturer's recommendations.

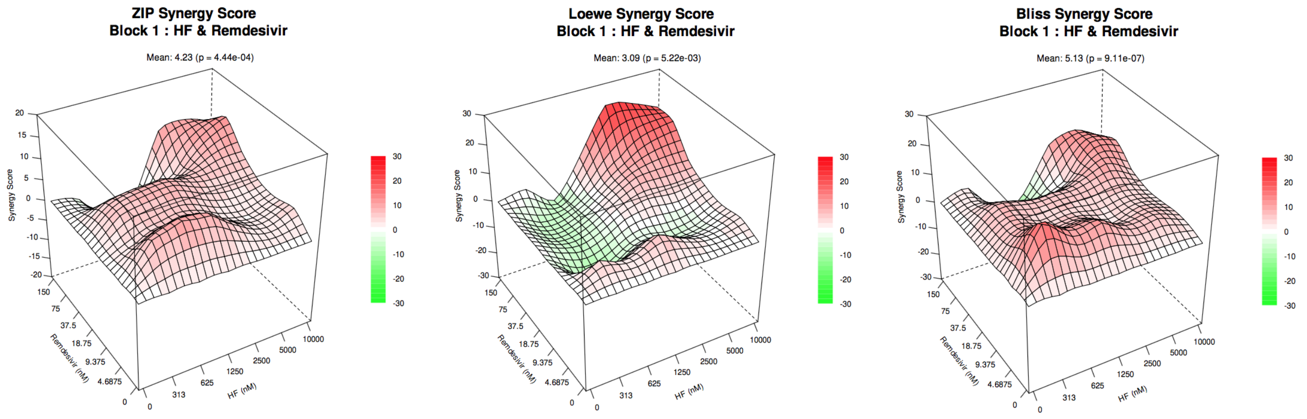

Supplementary Figure 5. Combination of hyperforin and remdesivir.
Heatmaps obtained with SynergyFinderPlus with ZIP, Loewe and Bliss models.

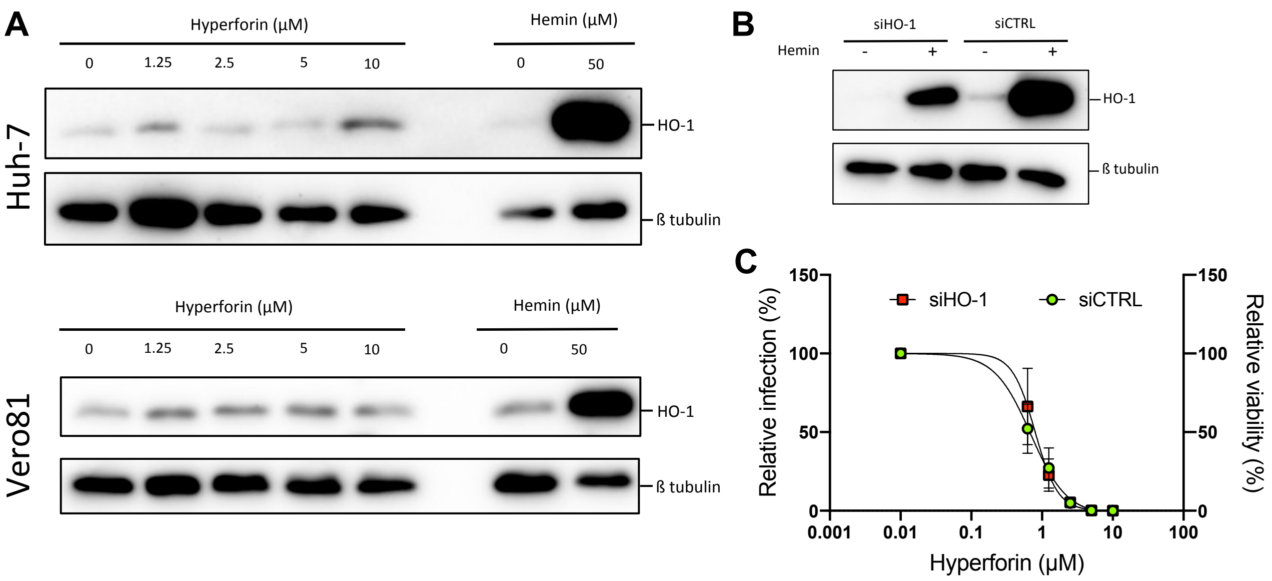

Supplementary Figure 6. The antiviral activity of hyperforin is not linked with HO-1 pathway.
(**A**) Huh-7 cells or Vero-81 cells were treated with increasing concentrations of hyperforin or with 50 µM hemin. HO-1 expression was then studied by Western Blot. (**B**) Huh-7 cells were treated with siRNA targeting HO-1 (siHO-1) or control siRNA (siCTRL). 24 h later, cells were treated or not with 50 µM hemin. HO-1 expression was then studied by Western Blot. (**C)** Dose-response assays of hyperforin against HCoV-229E were performed in presence of siHO-1 or siCTRL. The luciferase activity was measured after 24 h of incubation. Western Blots were representative for 3 independent experiments. Data were presented as means +/- SEM of 3 independent experiments performed in triplicates.

2.2 Supplementary Tables

Supplementary Table 1. Probes and primers used for RT-qPCR assays.

PRIME/PROBE	SEQUENCE
Mgene_229E_forward	5’-TTCCGACGTGCTCGAACTTT
Mgene_229E_reverse	5’-CCAACACGGTTGTGACAGTGA
Mgene_229E_probe	[6-FAM]-TCCTGAGGTCAATGCA-[BHQ1]
Egene_SARS2_forward	5’-ACAGGTACGTTAATAGTTAATAGCGT
Egene_SARS2_reverse	5’-ATATTGCAGCAGTACGCACACA
Egene_SARS2_probe	[6-FAM]-ACACTAGCCATCCTTACTGCGCTTCG-[MGB]
